# Dodging COVID-19 infection: low expression and localization of ACE2 and TMPRSS2 in multiple donor-derived lines of human umbilical cord-derived mesenchymal stem cells

**DOI:** 10.1186/s12967-021-02813-6

**Published:** 2021-04-14

**Authors:** Jonathan J. Hernandez, Doyle E. Beaty, Logan L. Fruhwirth, Ana P. Lopes Chaves, Neil H. Riordan

**Affiliations:** 1 Aidan Research and Consulting LLC, 11496 Luna Rd, suite 1100, Farmers Branch, TX 75234 USA; 2 Medistem Inc Panama, Ciudad del Saber, Edif. 221/Clayton, Panama, Republic of Panama

**Keywords:** ACE2 expression, TMPRSS2 expression, COVID-19, SARS-Cov2, Mesenchymal stem cells, HUC-MSC

## Abstract

**Background:**

Mesenchymal stem cells derived from human umbilical cord (hUC-MSCs) have immunomodulatory properties that are of interest to treat novel coronavirus disease 2019 (COVID-19). Leng et al. recently reported that hUC-MSCs derived from one donor negatively expressed Angiotensin-Converting Enzyme 2 (ACE2), a key protein for viral infection along with Transmembrane Serine Protease 2 (TMPRSS2). The purpose of this study was to quantify the expression of ACE2 and TMPRSS2 in hUC-MSCs lots derived from multiple donors using molecular-based techniques in order to demonstrate their inability to be a host to SARS-CoV-2.

**Methods:**

Expression of ACE2 and TMPRSS2 was analyzed in 24 lots of hUC-MSCs derived from Wharton's jelly via quantitative polymerase chain reaction (qPCR), Western Blot, immunofluorescence and flow cytometry using 24 different donors.

**Results:**

hUC-MSCs had significantly lower ACE2 (p = 0.002) and TMPRSS2 (p = 0.008) expression compared with human lung tissue homogenates in Western blot analyses. Little to no expression of ACE2 was observed in hUC-MSC by qPCR, and they were not observable with immunofluorescence in hUC-MSCs cell membranes. A negative ACE2 and TMPRSS2 population percentage of 95.3% ± 15.55 was obtained for hUC-MSCs via flow cytometry, with only 4.6% ACE2 and 29.5% TMPRSS2 observable positive populations.

**Conclusions:**

We have demonstrated negative expression of ACE2 and low expression of TMPRSS2 in 24 lots of hUC-MSCs. This has crucial implications for the design of future therapeutic options for COVID-19, since hUC-MSCs would have the ability to “dodge” viral infection to exert their immunomodulatory effects.

**Supplementary Information:**

The online version contains supplementary material available at 10.1186/s12967-021-02813-6.

## Background

With the worsening of the public health emergency caused by the global spread of the novel coronavirus disease 2019 (COVID-19) comes a pressing need to understand the molecular mechanisms driving the infection within the body [1]. The spike glycoprotein of SARS-CoV-2, the virus responsible for COVID-19, allows for entry into cells via human angiotensin converting enzyme II (ACE2) once primed by the cellular serine protease TMPRSS2 [2–4]. Therefore, cell membrane ACE2 and TMPRSS2 are integral components of viral transmission and spread [2].

A hyperinduction of inflammatory cytokines and chemokines such as interleukin (IL)-2R, IL-6, IL-8, IL-10, and tumor necrosis factor (TNF)-α among others, occurs in the lungs of critical patients with COVID-19 pneumonia: a “cytokine storm” [5–7]. Cytokine storms can result in pulmonary edema, air exchange dysfunction, acute respiratory distress syndrome, acute heart failure, secondary infections, and death [8]. In light of current treatments for severe cases of COVID-19 having had mixed results or serious adverse events [9–11], there remains a global interest to find safe and effective avenues of treatment.

Mesenchymal stem cells (MSCs) [12, 13] exert anti-inflammatory [14–16], anti-bacterial, anti-protozoan and anti-viral [17–20] effects making them a possible treatment for COVID-19-related complications [21–24]. MSCs produce several types of cytokines via paracrine secretion or have direct interactions with immune cells, resulting in immunomodulation [25]. MSCs derived from the Wharton Jelly tissue of the human umbilical cord (hUC-MSCs) possess a high capacity for proliferation and immunomodulation [26–28]. In a study of 150 people with confirmed cases of COVID-19, levels of the inflammatory cytokine IL-6 were significantly higher in non-survivors than in survivors of the disease [29]. Furthermore, the upregulation of IL-6 in fatal cases of COVID-19 indicates that mortality may be driven by hyperinflammation [30]. UC-MSCs have been used to modulate IL-6 in the body: for example, the levels of IL-6 decreased around 50% three months after treatment in a study of 172 patients with rheumatoid arthritis [31].

hUC-MSCs were used experimentally to treat seven patients with confirmed COVID-19 pneumonia in China [32]. The pulmonary function of all patients significantly improved within two days of hUC-MSC transplantation and levels of TNF-α were significantly reduced. Additionally, the gene expression profile in this study showed that MSCs were ACE2- and TMPRSS2-. However, the hUC-MSCs used for treatment were derived from only one donor, representing a limitation for a broader extrapolation of these results.

In this study, we investigated the expression of ACE2 and TMPRSS2 in hUC-MSCs lines derived from different donors using quantitative polymerase chain reaction (qPCR), Western Blot, immunofluorescence and flow cytometry. Human pulmonary alveolar cells type I, human lung homogenates, human lung RNA, and one lot of hUC-MSCs transfected with an ACE2 expression plasmid were used as controls.

## Methods

### Cell culture

Primary Human Bronchial Epithelial Cells (HBEpC) were obtained from PromoCell (Cat. # C-12640). HBEpC were isolated from the surface epithelium of human bronchi and stain positive for cytokeratin. HBEpC are useful for investigating the function and pathology of the respiratory system and were used as controls. Human pulmonary alveolar epithelial cells type I (AT1) were obtained from AcceGen Biotechnology (Cat. # ABC-TC3770) and cultured following the manufacturer’s guidelines and were used as controls.

Twenty-four lots of culture expanded human hUC-MSCs were utilized in this study, isolated from human umbilical cord tissue from normal, healthy births, voluntarily donated with a fully executed informed consent form. Sixteen of those lots were obtained from a biotechnology company that manufactures hUC-MSCs for use in clinical trials (Medistem Panama, City of Knowledge of the Republic of Panama, a laboratory licensed by the Panamanian Ministry of Health, following Good Tissue Practices 21 CFR 1271). The cell lots were passage 5 with a doubling time of < 24 h and were received frozen in dry shippers and stored at − 150 °C until studied. Manufacturing methodology is described in detail in publications of clinical trials that used these hUC-MSCs for treatment [33, 34].

For the other eight lots, umbilical cords were obtained from an FDA-registered tissue bank licensed by the AATB in the United States (FEI 300367004), according to current Good Tissue Practices from healthy, full-term, scheduled uncomplicated C-sections. Written informed consent was obtained from the donors. The isolation, selection and culture were performed by Aidan Research and Consulting LLC for research purposes only. The isolation process was done using the Umbilical Cord Dissociation Kit, human (Miltenyi Biotec Cat. # 130-105-737) following the manufacturer’s guidelines. Cells were expanded through passage 5 and used for the measurements reported here.

The intended use of all cells was for research only and not for clinical use in human participants. Researchers did not have access to any identifying information of biospecimens. All cell lots used in this study met release criteria, namely: 75% viability and > 95% positive for CD90, CD73, CD105 cell surface markers as determined by flow cytometry (Additional file 1: Figure S2). These 24 lots were all used as samples for the subsequent experiments, and one of the Aidan Research and Consulting lots was transfected and used as a positive control.

### Trilineage differentiation

To determine the multipotency capacity of hUC-MSCs, adipogenic, osteogenic, and chondrogenic differentiation experiments were performed following the directed instructions of PromoCell MSC Differentiation Media (Cat. # 28016, 28012, 28014, 28013, 28015). For adipogenic differentiation, lipid droplets of the resultant differentiated cells were detected using Oil red staining (Sigma-Aldrich, Cat. # MAK194). To assess osteogenic differentiation, Alizarin Red S staining (Sigma-Aldrich, Cat. # TMS-008) was performed for the calcium-rich extracellular matrix. For chondrogenic differentiation, after 3 weeks a chondrogenic pellet was harvested and fixed in 4% paraformaldehyde (PFA) stained with Alcian Blue (Sigma-Aldrich, Cat. # 1016470500) (Additional file 1: Figure S2b).

### Transient transfection

For transfection, 6000 hUC-MSCs per cm^2^ were plated in 100 cm^2^ cell culture dishes. Once they reached 70% of confluency, 20 µg of the ACE2 and TMPRSS2 dual expression vector pDUO2-hACE2-TMPRSS2 (InvivoGen Cat. # pduo2-hace2tpsa) was transfected using Lipofectamine™ Stem Transfection Reagent (Thermo Fisher Scientific Cat. # STEM00015) following the manufacturer’s instructions. Forty-eight hours after transfection cells were either only fixated using Image-iT™ Fixation Kit (Thermo Fisher Scientific Cat. # R37602) for imaging or lysed using RIPA buffer (Thermo Fisher Scientific Cat. # 89,901) with 1X protease and phosphatase inhibitor (Thermo Fisher Scientific Cat. # 78,444) for Western Blot analysis.

### Protein preparation and western blot

Human lung homogenates were purchased from the OriGene tissue bank (CP565585, CP565542, CP565577, CP565443, CP565542 and CP565586). Whole protein was obtained by sonication of hUC-MSCs (transfected and non-transfected), AT1 and HBEpC scrapped with RIPA buffer supplemented with 1X protease and phosphatase inhibitor and quantified using the Pierce™ Rapid Gold BCA Protein Assay Kit (Thermo Fisher Scientific Cat. # A53226). 25 µg of protein was added to 4 × LDS loading buffer and incubated at 50 °C for 5 min. SDS-PAGE was performed with Criterion TGX Stain-free 4–20% Gel (Bio-Rad Cat. # 5678093) and transferred to a PVDF membrane using the iBlot™ 2 Gel Transfer Device (Thermo Fisher Scientific Cat. # IB21001). Membranes were blocked for 1 h in StartingBlock™ T20 (TBS) Blocking Buffer (Thermo Fisher Scientific Cat. # 37543) at room temperature and incubated overnight at 4 °C with 1:500 Rabbit anti-ACE2 (Thermo Fisher Scientific Cat. # MA5-32307), 1:500 primary antibody TMPRSS2 made in rabbit (Abcam Cat. # ab92323), and 1 h at room temperature with 1:10,000 Mouse anti-GAPDH (Millipore, MAB374). Membranes were incubated in secondary antibodies, 1:5000 Alexa Fluor 680 goat anti-rabbit (Thermo Fisher Scientific Cat. # A21076) and 1:5000 Alexa Fluor 488 donkey anti-mouse (Thermo Fisher Scientific Cat. # A21202), for one hour at room temperature. Detection of relevant proteins and images were taken using iBright FL1500 Imaging System (Thermo Fisher Scientific). For relative quantification, the volume intensity of the bands was obtained using iBright software. The relative expression was calculated by dividing the values to GAPDH.

### Quantitative real-time polymerase chain reaction (qPCR)

Total RNA was isolated from cells using the Trizol™ Plus RNA Purification Kit (Thermo Fisher Scientific Cat. # 12183555) and DNA was removed using the TURBO DNA-free Kit Thermo Fisher Scientific Cat. # AM1907 from all hUC-MSC lots, AT1 and HBEpC. Additionally, RNA isolated from human lung tissues (OriGene Technologies; Cat. #: CR559346, CR559185, CR560789, CR562469 and CR561266) was included as a positive control (n = 5). All RNA extractions were then quantified using a Varioskan LUX™ (Thermo Fisher), and their integrity was checked using a 1% E-Gel™ EX Agarose Gel (Thermo Fisher Scientific Cat. # G401001). Subsequently, 1 µg of purified RNA was reverse-transcribed to cDNA using the iScript™ cDNA Synthesis Kit (Biorad Cat. # 1708890) following the manufacturer protocol. Then, 20 ng of cDNA was amplified by qPCR using the TaqMan™ Fast Advanced Master Mix along with TaqMan™ Gene Expression Assays for ACE2 (Hs01085333_m1), TMPRSS2 (Hs01120965_m1), and for the reference gene PPIA (Hs99999904_m1). All qPCR reactions were performed in triplicates on a QuantStudio™ 3 Real-Time PCR System (Thermo Fisher Scientific). Raw cycle thresholds values were calculated using the QuantStudio™ Design and Analysis Software v.1.5.1 using automatic baseline settings and a threshold of 0.3. The relative expression of genes of interest was normalized to the expression of PPIA. A Mann–Whitney Rank Sum Test was used to calculate statistically significant differences between expression of ACE2 and TMPRSS2 in human lung RNA, AT1 cells, HBEpC and hUC-MSCs.

### Immunofluorescence

Cells were plated on 12-well glass-bottomed MatTek plates (P12G-1.5-14-F) and only fixed using an Image-iT™ Fixation Kit (Thermo Fisher Scientific Cat. #R37602) following the manufacturer instructions. Primary antibody ACE2 anti-mouse (Thermo Fisher Scientific Cat. # MA5-31395), in a dilution 1:50, was added to the indicated wells and left overnight at 4 °C. The primary antibody TMPRSS2 made in rabbit (Abcam Cat. # ab92323), in a dilution 1:50 was used and incubated overnight at 4 °C. Secondary antibodies Alexa Fluor 488 donkey anti-mouse (Thermo Fisher Scientific Cat. # A21202) and Cyanine3 goat anti-rabbit (Thermo Fisher Scientific Cat. # A10520), in a dilution 1:2000, were added to the wells and incubated for one hour at room temperature. Coverslips were mounted onto the slides using Prolong DAPI (Thermo Fisher Scientific Cat. # P36935) and images were taken using a Lionheart FX automated microscope (BioTek) using the same exposure settings in all the groups.

### Flow cytometry

For the immunophenotype of hUC-MSCs [35], when colony-forming units reached 70% of confluency, hUC-MSCs were selected using the MSC Phenotyping Kit, human (Miltenyi Biotec Cat. # 130–125-285). All single cells negative for CD34, CD45, CD11b or CD14, CD19, and HLA-DR (all in PerCP channel), as well as > 95% positive cells for CD73, CD90 and CD105, were sorted using SH800 cell sorter (Sony Biotech) (Additional file 1: Figure S2).

Single cell suspensions of 24 hUC-MSC lots, one HBEpC and one AT1 were single stained with primary antibody TMPRSS2 made in rabbit (Abcam Cat. # ab92323) or Rabbit anti-ACE2 (InvitrogenThermo Fisher Scientific, Cat. # MA5-32307) with a secondary PE-Cy5.5 goat anti-rabbit antibody (InvitrogenThermo Fisher Scientific, Cat. # L42018). Non-stained controls and Non-Specific-Binding (stained only with PE-Cy5.5 goat anti-rabbit) were used to identify false positive populations and these values were subtracted from all the sample groups. The cells were resuspended in 300 µL of sorting buffer (0.05% FBS in PBS) and analyzed using the flow cytometry functions on a Sony SH800S Cell Sorter (SONY Biotechnology).

### Statistical analysis

SigmaPlot 12.5 (Systat Software) was used for all statistical analyses. Means and standard errors of the means were calculated for all relevant quantities. One-way ANOVA (Mann–Whitney Rank Sum Test) was used for all comparisons. A p-value ≤ 0.05 was taken as the level for statistical significance.

## Results

### Western blot reveals little expression of ACE2and low levels of TMPRSS2 and in hUC-MSCs cell lysates

**Fig. 1:**
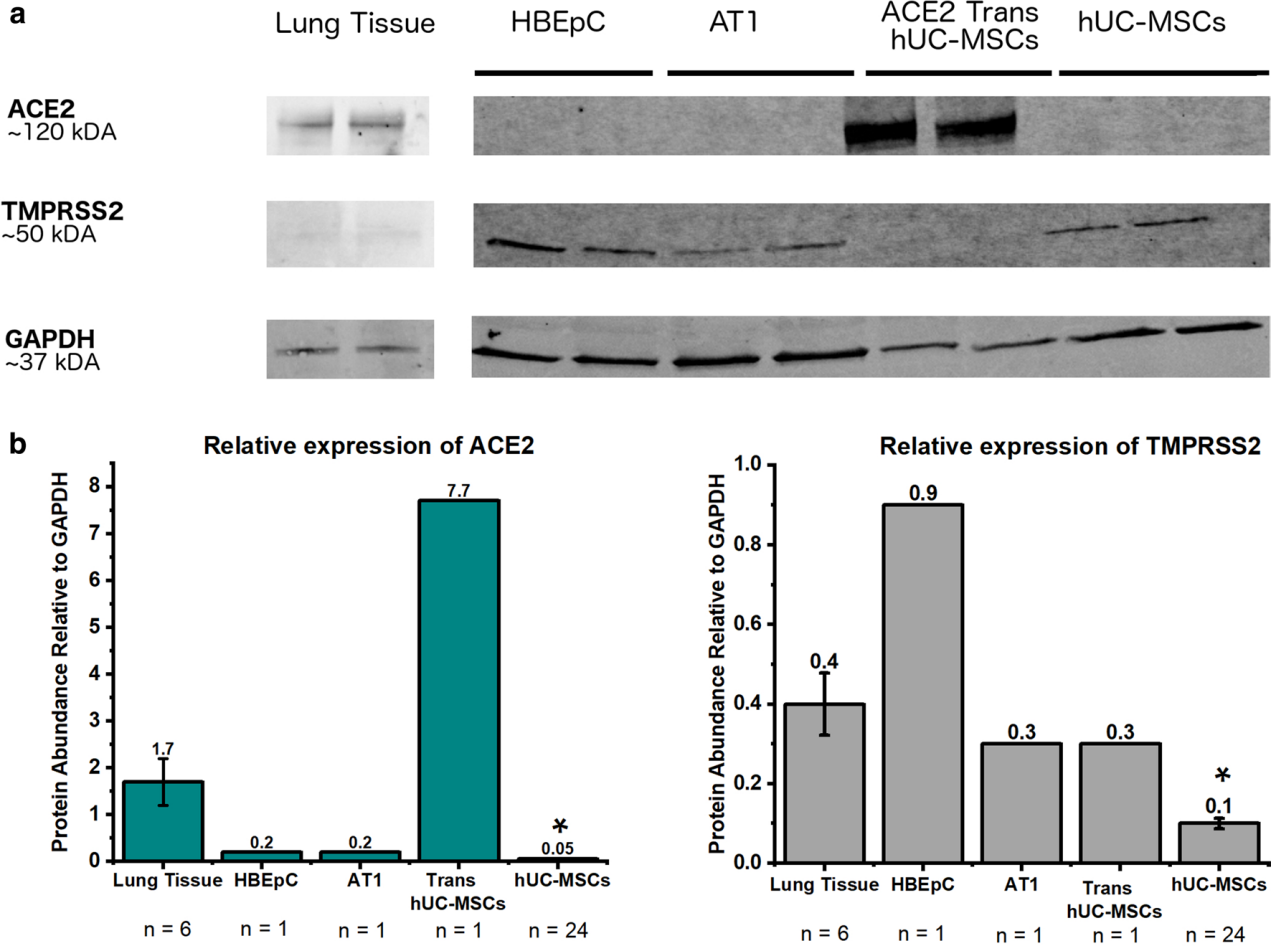
**a** Representative blots. **b** Expression levels of ACE2 and TMPRSS2 relative to GAPDH in protein homogenates from human lung, primary human bronchial epithelial cells (HBEpC), human pulmonary alveolar type 1 cells (AT1), transfected hUC-MSCs and hUC-MSCs. hUC-MSCs had significantly lower expression of ACE2 (p = 0.002) and TMPRSS2 (p = 0.008) compared to lung homogenates. The coefficient of variance for lung tissue was 46.9% and 52.3% for for hUC-MSC

hUC-MSCs (n = 24) had significantly lower (p = 0.002) ACE2 expression relative to GAPDH compared with lung tissue homogenates (n = 6) in Western blot analyses (Fig. 1). A trend for lower expression of ACE2 in hUC-MSCs was observed when compared to hUC-MSCs transfected with ACE2 expressing plasmid (n = 1). HEBpC and AT1 (n = 1) also showed low expression of ACE2 but higher expression of TMPRSS2. TMPRSS2 levels in hUC-MSCs were significantly lower when compared to lung tissue (p = 0.008). Although expression was low, TMPRSS2 levels were variable between hUC-MSCs from different donors (coefficient of variation 52.3%). ACE2 bands were observed to have a molecular weight of 120 kDa, while TMPRSS2 showed a thin band around 50 kDa. Expression of both proteins was normalized to GAPDH, which was observed at 37 kDa.

### Low gene expression of ACE2 and TMPRSS2

**Fig. 2:**
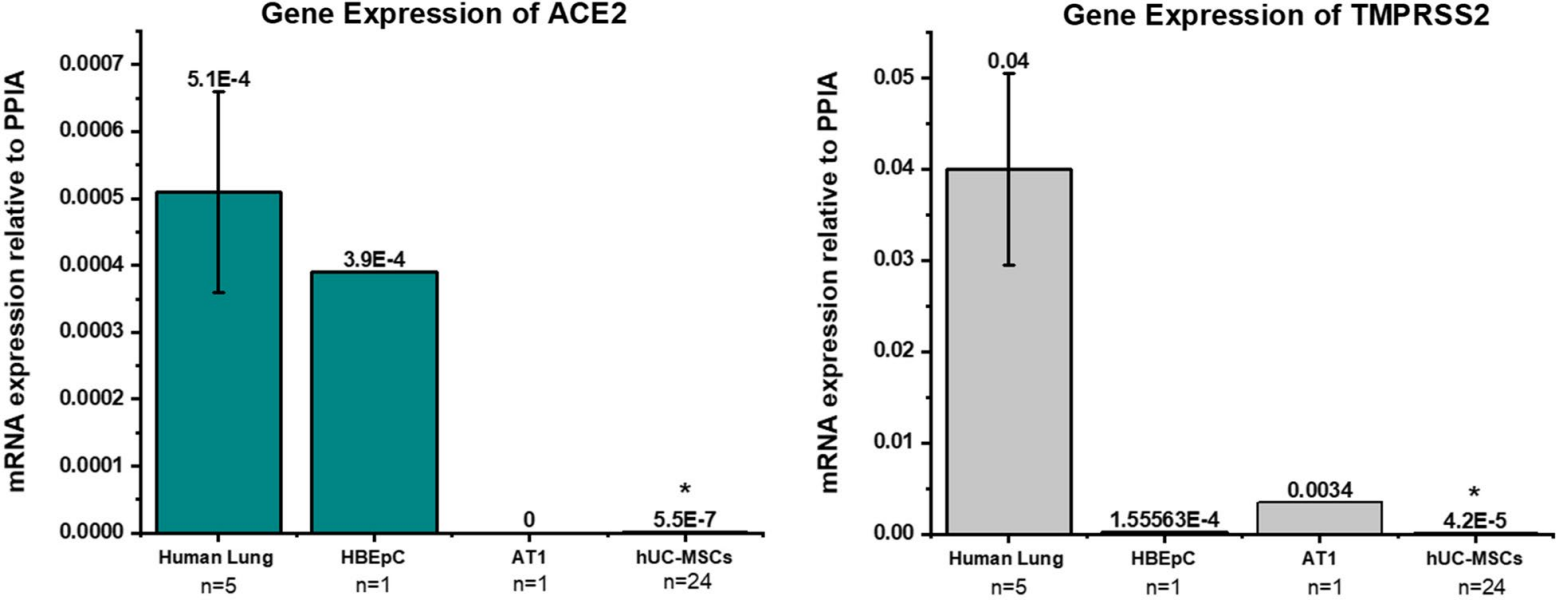
ACE2 and TMPRSS2 expression in human lung-derived tissue, primary human bronchial epithelial cells HBEpC, human pulmonary alveolar type 1 cells (AT1), and hUC-MSCs on qPCR. The relative expression of the genes of interest was normalized to the expression of PPIA. Human lung RNA, AT1 and HBEpC were used as controls (tissue and single cells). The expression of ACE2 and TMPRSS2 was significantly lower in hUC-MSCs compared to human lung tissue (*P* ≤ 0.001* for both), and the coefficient of variance of TMPRSS2 was 69.54%

Expression of ACE2 and TMPRSS2 was observed in all human lung RNA samples (n = 5) while little to no expression was observed in hUC-MSC (n = 24), AT1 (n = 1) and HBEpC observed on qPCR. AT1 expressed less TMPRSS2 than the lung tissue and did not express ACE2. For each sample, relative expression of the genes was averaged to represent differences between the sample groups. Significant differences in the expression of ACE2 and TMPRSS2 were observed between the human lung RNA and hUC-MSCs (*p* ≤ 0.001 for both) according to the Mann–Whitney Rank Sum Test (Fig. 2). Overall, expression of TMPRSS2 was higher in all groups. TMPRSS2 levels were variable between hUC-MSCs from different donors (qPCR coefficient of variation 69.54%).

### ACE2 is not localized in the cell membrane of hUC-MSCs

**Fig. 3:**
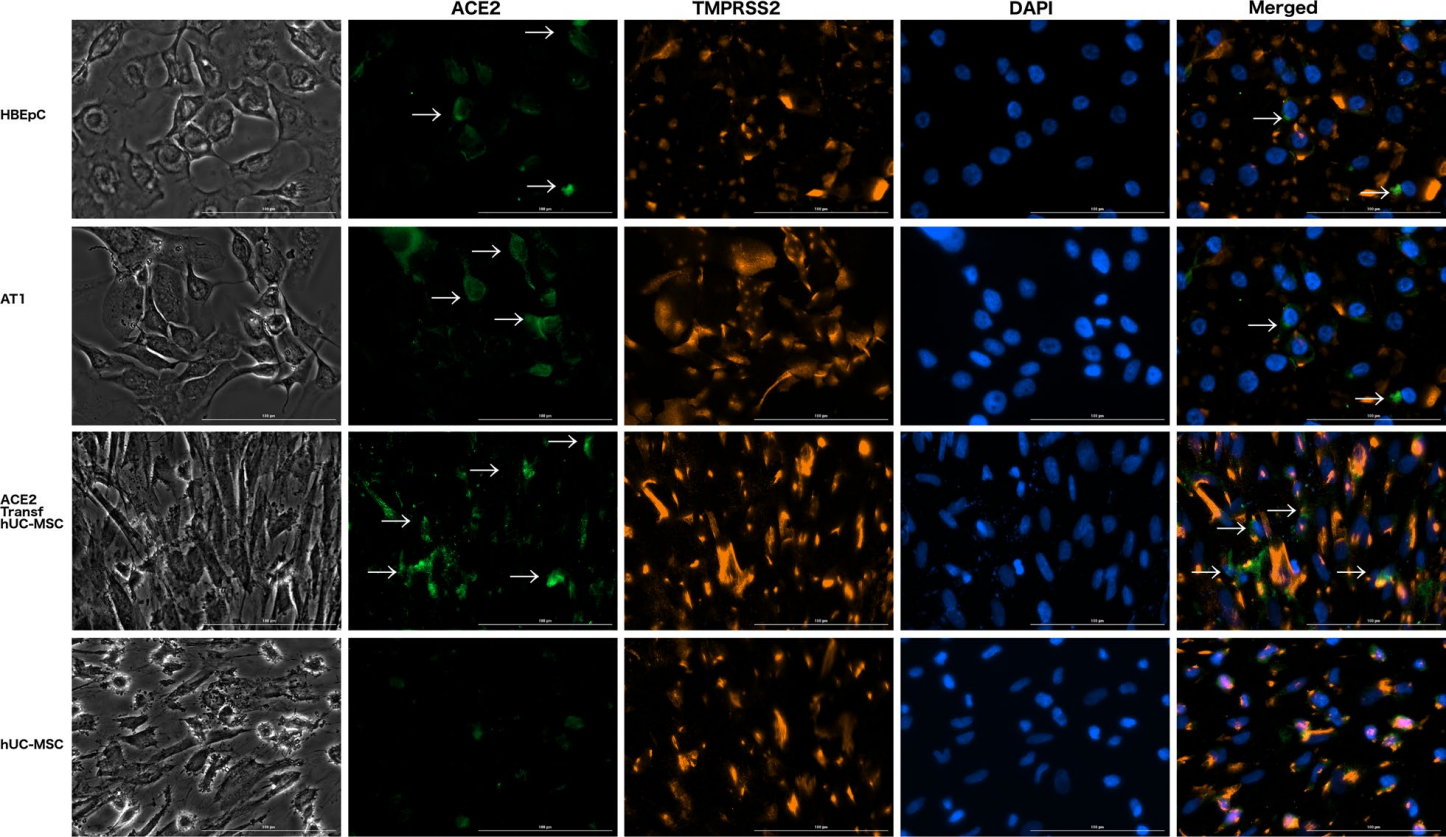
Immunofluorescent staining of ACE2, TMPRSS2 and nuclei for primary human bronchial epithelial cells (HBEpC), human pulmonary alveolar type 1 cells (AT1), transfected hUC-MSCs and hUC-MSCs. White arrows indicate ACE2 expression

Fluorescence microscopy was conducted to determine the presence and localization of ACE2 and TMPRSS2 in the membrane of the cells (HBEpC, AT1, hUC-MSC transfected with pDUO2-hACE2-TMPRSS2 plasmid, and hUC-MSCs). ACE2 was not observed in the cell membrane of hUC-MSCs (Fig. 3). However, the expression of TMPRSS2 was observed in the cell membrane in all the groups. hUC-MSCs transfected with the plasmid restored the expression of ACE2 in the cell membrane (Fig. 3).

### Most hUC-MSCs populations are negative for ACE2 and TMPRSS2

**Fig. 4:**
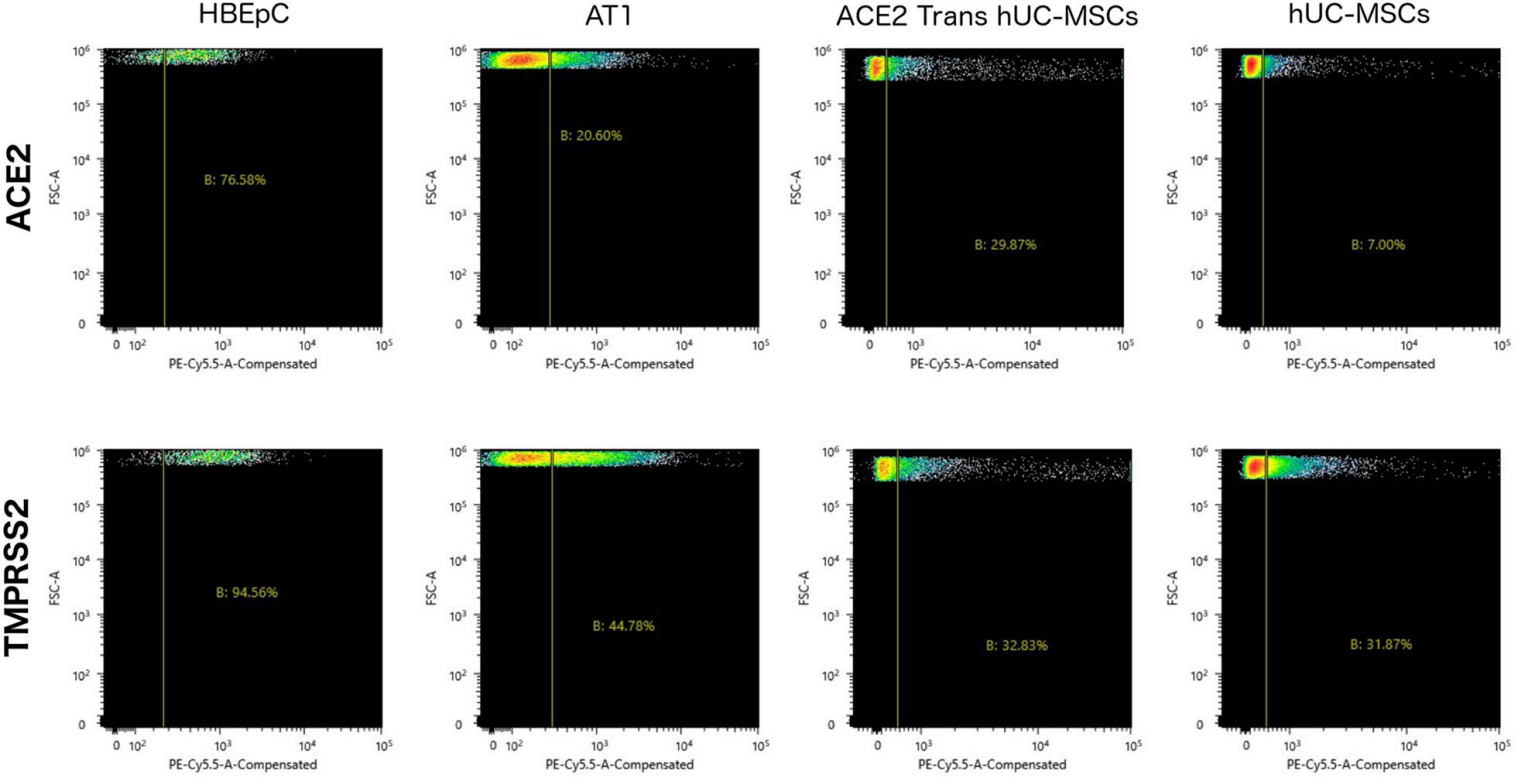
Flow cytometry for ACE2 and TMPRSS2 of primary human bronchial epithelial cells (HBEpC), human pulmonary alveolar type 1 cells (AT1), transfected hUC-MSCs and hUC-MSCs

Flow cytometry of cell populations of HBEpC, AT1, transfected hUC-MSC and hUC-MSC were gated to observe single positive populations, as well as hUC-MSC with non-specific binding (NSB) to discard false positive cell populations from the test group (Fig. 4). The HBEpC population percentages were 19.7% NSB positive, 56.9% ACE2 positive, 74.8% TMPRSS2 positive. The AT1 population percentages were 4.4% NSB positive, 16.1% ACE2 positive, 40.3% TMPRSS2 positive. The transfected hUC-MSC population percentages were NSB 7.2% positive, 22.6% ACE2 positive, 25.59% TMPRSS2 positive. The hUC-MSC population percentages were NSB 2.25% positive, 4.6% ACE2 positive, 29.6% TMPRSS2 positive.

## Discussion

Mesenchymal stem cells (MSCs) are currently being considered as a potential treatment of COVID-19 and its associated complications [22, 23]. Given that ACE2 and TMPRSS2 are crucially involved in transmission and spread of the virus, we sought to investigate their expression in hUC-MSCs. In this study, we have demonstrated the negative expression of ACE2 and low expression of TMPRSS2 in 24 lots of hUC-MSCs derived from different donors. We investigated levels of gene expression and localization of the protein in the cells via different methods: gene expression was not found using qPCR and Western Blot in any of the 24 lots of hUC-MSCs compared to the positive control of human lung RNA; ACE2 was not detected in the hUC-MSCs membrane with immunofluorescence, and flow cytometry revealed that only 4.6% and 29.6% of MSCs positively expressed ACE2 and TMPRSS2, respectively. This broadens the novel findings of Leng et al. regarding the expression of these specific genes in hUC-MSCs [32] to a larger number of cell lots derived from different donors.

However, we clearly observed differences in the expression of TMPRSS2 at the gene expression level (qPCR coefficient of variation 69.54%) and at the protein level (Western Blot coefficient of variation 52.3%) and localization in different cell types that do not correlate with the gene expression results reported in Leng et al.'s work and in another recent study [36]. In addition to protein expression, localization, and cell population, the choice of antibody was of particular importance for the design of this experiment: certain antibodies were suitable for e.g. Western blot experiments, but unsuitable for immunofluorescence. Choosing rabbit anti-ACE2 (# MA5-32307) allowed us to use a single antibody for Western blot, immunofluorescence, and flow cytometry, and levels of expression were corroborated with all techniques. Furthermore, levels of gene expression may vary depending on the tissue from which MSCs are derived: ACE2 levels in hematopoietic induced pluripotent stem cells (iPSCs), for example, would not correspond to those levels found in Wharton's jelly derived hUC-MSCs.

It is critical, when reporting in-vitro levels of expression of a particular protein, to correctly assess this with several techniques instead of relying solely on gene expression or mRNA levels. This is particularly important when findings are intended to serve as a starting point for clinical applications. Levels in mRNA do not always correlate with levels in protein content [37–40]. A single method of measurement may therefore not be enough to fully investigate expression levels; we are here proposing that qPCR, Western blot, immunofluorescence and flow cytometry are to be used as complements of each other in detecting ACE2 and TMPRSS2 expression to have a well-rounded overview at the cellular level.

**Fig. 5:**
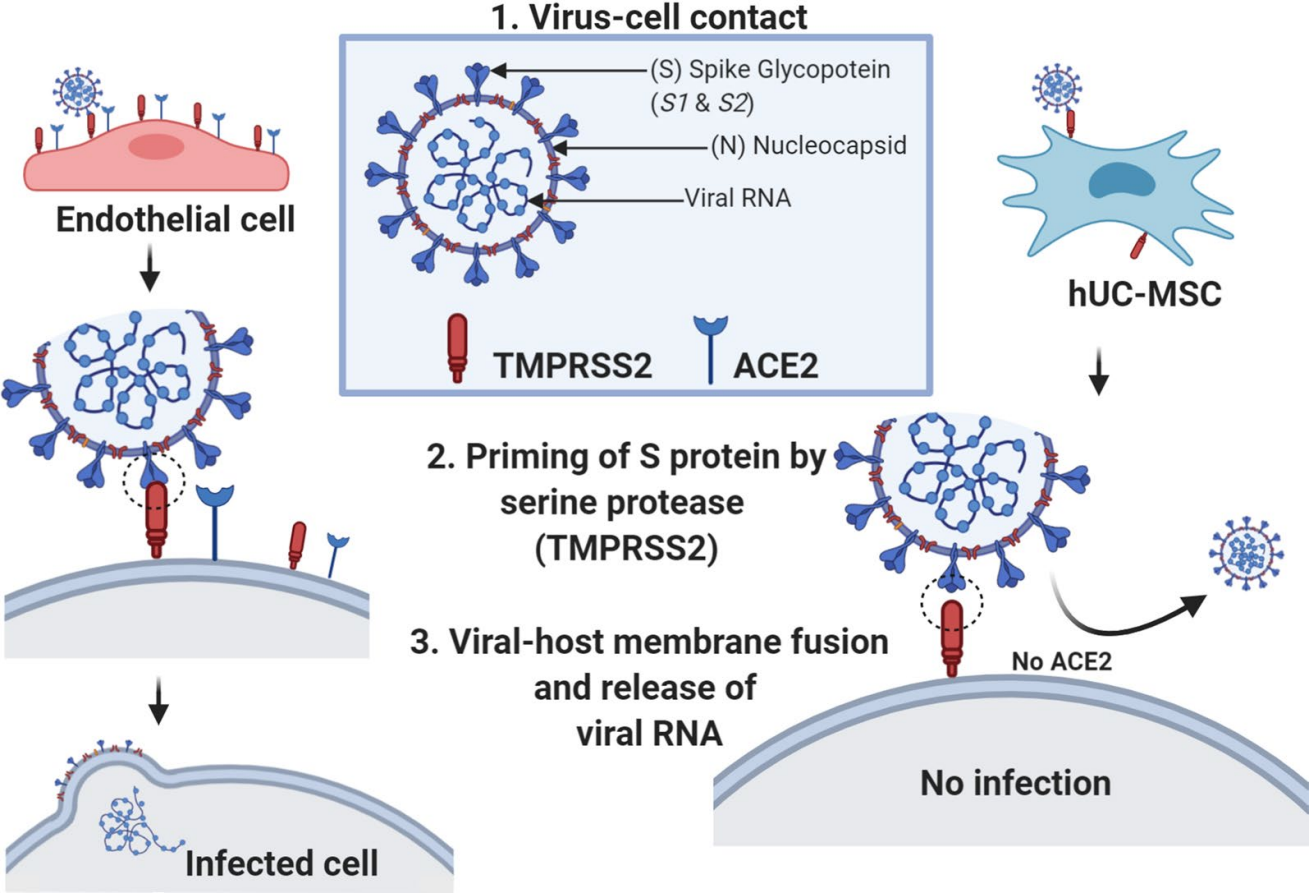
Left: mechanisms of infection of SARS-COV-2 in a common host cell, positively expressing ACE2 and TMPRSS2. Right: mechanisms of infection of SARS-COV-2 in a mesenchymal stem cell, negatively expressing ACE2 and TMPRSS2. Figure created with BioRender.com

Lower expression or lack of expression of ACE2 and TMPRSS2 could have crucial implications for the design of future therapeutic options for COVID-19. Since the virus engages ACE2 as the entry receptor [41] and employs the cellular serine protease TMPRSS2 for the spike glycoprotein S priming [42, 43], ACE2^−^ and low TMPRSS2 expressing cells should likely remain uninfected by the virus (Fig. 5). Demonstrating that hUC-MSCs do not appreciatively express ACE2 and express low levels of TMPRSS2, coupled with their immunomodulatory, anti-inflammatory and antimicrobial properties, could position them as a viable treatment option. After being administered to a COVID-19 patient, a large majority of hUC-MSCs should be able to exert their therapeutic action via the secretion of anti-inflammatory molecules while “dodging” the virus (Fig. 5). To date, MSCs have been used to treat pulmonary conditions such as idiopathic pulmonary fibrosis, acute respiratory distress syndrome and chronic obstructive pulmonary disease [14]. MSC-derived exosomes were also able to revert pulmonary fibrosis in a mouse model [44]. Likewise, trophic factors secreted by MSCs (MTF), which can be administered by inhalation, have shown therapeutic benefits for pulmonary disease [45, 46]. In light of this, international trials are currently ongoing to treat COVID-19 with mesenchymal stem cells derived from a variety of tissues [47].

The intent of investigating the expression of ACE2 and TMPRSS2 was to determine the likelihood of hUC-MSCs becoming infected by SARs-Cov-2, as it would be counterproductive to supply the virus with a fresh influx of susceptible cells. Quality control is of utmost importance when considering treatment with MSCs for COVID-19; in addition to investigating the infectivity potential of MSCs, attention should also be given to concerns about the variability of TF/CD142 expression among cells lots, which may trigger blood clotting and thromboembolism in this hypercoagulable pathology [48]. High expression of TF/CD142 on cells used in the treatment of COVID-19 could lead to dire consequences if the cells enhanced the already present pro-thrombotic effects of the viral infection itself [49, 50].

The extent of the ability of hUC-MSCs to mitigate the devastating cytokine storm observed in critically ill patients should be a next avenue in further research, particularly to ascertain which cytokines can be induced back to their normal levels in the presence of hUC-MSC secretions—this could lead to more targeted therapeutics. Since the levels of inflammatory cytokines and chemokines have been reported to be abnormally elevated in critically ill COVID-19 patients [5, 32], the immunomodulatory effects of hUC-MSCs on these cytokines, and particularly in a setting of simultaneous overexpression, should be investigated promptly. Caution should be exerted, however, to administer hUC-MSCs outside of the clinically demonstrable cytokine storm, as MSC immunomodulatory effects could paradoxically aid the virus in earlier phases of the infection.

Furthermore, AGTR2 could be a third marker of interest in addition to ACE2 and TMPRSS2, since its affinity to the spike protein of the virus appears to be higher than ACE2 [51]. Over the course of this study, we began investigating AGTR2 expression in hUC-MSCs compared to controls, finding negative expression (data not shown). We hope to expand upon these findings in future experiments.

Multiple repositories were contacted at the beginning of this study and alveolar cells type II were not available to us at the time, resulting in significant limitations for this study. The impossibility to obtain said alveolar cells for research in a reasonable timeframe meant choosing human lung and as pulmonary alveolar cells type I as controls in limited quantities. Alveolar cells type II would have been more ideal to replicate the more intrinsic mechanisms of SARS-CoV-2 infection, but we hope that these results may be extended to that type of cell when it becomes more readily available for research. Only twenty-four different donors were considered for this experiment, but we expect that these results could be replicated with a larger number of hUC-MSC lots. Finally, our current laboratory does not possess BSL-4 facilities that would allow culturing and testing the infection rate of SARS-CoV-2 in hUC-MSCs to verify our findings.

Levels of ACE2 expression are of interest for COVID-19 research, and a recent report has indicated low ACE2 expression levels in MSCs derived from different tissues, though it is unclear how many cell lines were used in that particular context [36]. More recently, Lazoni et al. have reported results from a small clinical trial where hUC-MSCs were used to safely treat COVID-19 patients [52]. We have studied twenty-four different hUC-MSCs cell lines and have determined levels ACE2 and TMPRSS2 therein with several techniques, leading to our robust conclusion of no ACE2 expression and low TMPRSS2 infection in Wharton's jelly-derived hUC-MSCs. By having used hUC-MSC lots derived from multiple donors, we have established that there is no ACE2 expression in hUC-MSCs, while TMPRSS2 expression could be donor-dependent. This provides a reasonable basis to support the evasion of infection by hUC-MSCs, making them an effective, non-vector, option for COVID-19 treatment, thereby supporting the findings of Avanzini et al. [53] and Leng et al. [32].

## Conclusions

hUC-MSCs have the potential to provide a safe, effective treatment for critically ill COVID-19 patients, which could help mitigate the devastating economic and public health consequences caused by the rapid worldwide spread of SARS-CoV-2. We have demonstrated negative expression of ACE2 and low expression of TMPRSS2, key proteins in the SARS-Cov2 infection process, in twenty-four lots of hUC-MSCs, and we hope that these results will encourage further research into hUC-MSCs for the treatment of the inflammatory effects of COVID-19 infection.

## Supplementary Information

**Additional file 1: Figure S1.** a) Full length blots of representative samples HBEpc, AT1, ACE2 Trans hUC-MSCs. b) Full length blots of representative sample Lung tissue. iBright™ Prestained Protein Ladder (LC5615) was used as the molecular weight marker. **Figure S2.** Immunophenotype and trilineage differentiation of hUC-MSCs lines. a) Representative figures of flow cytometry analysis showing lower percentages of cells positive for CD14, CD20, CD34 and CD45 (DUMP:PreCP) and higher percentages of CD73 (PE-Vio770), CD90 (FITC) and CD105 (PE). b) Representative staining. Multipotency/Trilineage differentiation of hUC-MSCs was assayed after culture in specific medium for the osteogenic, adipocyte and chondrogenic differentiation. Specific extracellular matrix components were stained using Alzarin Red for osteoblasts, adipocytes were detected with Oil Red coloration of lipid droplets, and chondrocytes with alcian Blue.

## Data Availability

The data that support the findings of this study are available from the corresponding author upon reasonable request.
