# Supplementary material for: Dodging COVID-19 infection: low expression and localization of ACE2 and TMPRSS2 in multiple donor-derived lines of human umbilical cord-derived mesenchymal stem cells
Source: J Transl Med. 2021 Apr 14;19:149. doi: 10.1186/s12967-021-02813-6 (PMC8045575; doi:10.1186/s12967-021-02813-6)
Supplement: Supplementary file 1 — Additional file 1: Figure S1. a) Full length blots of representative samples HBEpc, AT1, ACE2 Trans hUC-MSCs. b) Full length blots of representative sample Lung tissue. iBright™ Prestained Protein Ladder (LC5615) was used as the molecular weight marker. Figure S2. Immunophenotype and trilineage differentiation of hUC-MSCs lines. a) Representative figures of flow cytometry analysis showing lower percentages of cells positive for CD14, CD20, CD34 and CD45 (DUMP:PreCP) and higher percentages of CD73 (PE-Vio770), CD90 (FITC) and CD105 (PE). b) Representative staining. Multipotency/Trilineage differentiation of hUC-MSCs was assayed after culture in specific medium for the osteogenic, adipocyte and chondrogenic differentiation. Specific extracellular matrix components were stained using Alzarin Red for osteoblasts, adipocytes were detected with Oil Red coloration of lipid droplets, and chondrocytes with alcian Blue. [file 12967_2021_2813_MOESM1_ESM.docx]

**Supplementary Information**

**Title: Dodging COVID-19 Infection: Low Expression and localization of ACE2 and TMPRSS2 in Different Donor-derived Lines of Human Umbilical Cord-derived Mesenchymal Stem Cells**

**Running title**: ACE2 and TMRPSS2 expression in hUC-MSCs

**Authors:**

Jonathan J Hernandez^1,2^, Doyle E Beaty^1^, Logan L Fruhwirth^1^, Ana P Lopes Chaves^1^, Neil H Riordan^1,2^

1. Aidan Research and Consulting LLC, 11496 Luna Rd, suite 1100, Farmers Branch, Texas 75234
2. Medistem Inc Panama, Ciudad del Saber, Edif. 221 / Clayton Panama, Rep. of Panama.

**Corresponding author:** Jonathan J Hernandez, PhD. jhernandez@aidanresearch.com

**Keywords**: ACE2 expression, TMPRSS2 expression, COVID-19, SARS-Cov2, mesenchymal stem cells, hUC-MSC


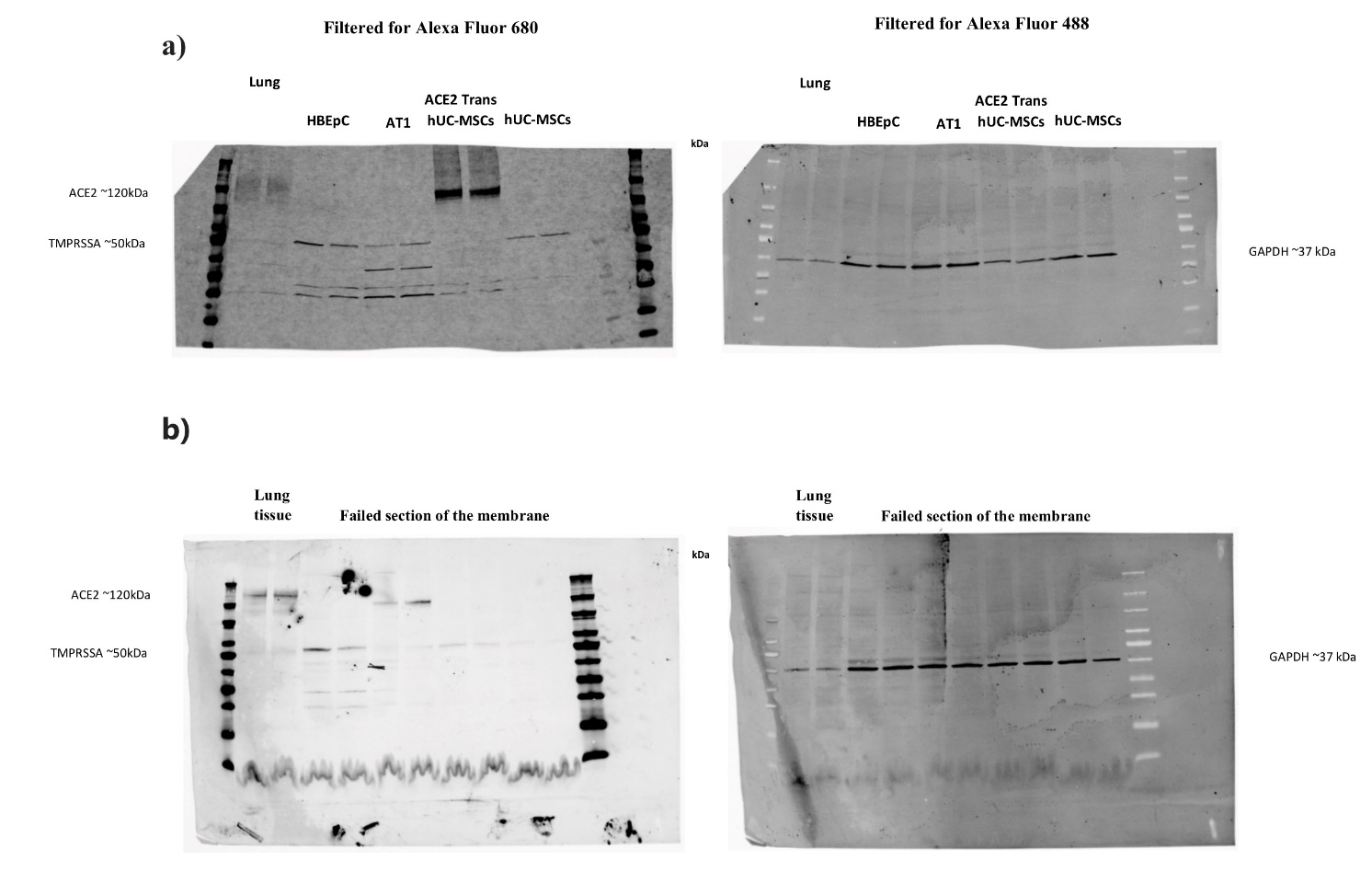
**Supplemental Figure 1.** a) Full length blots of representative samples HBEpc, AT1, ACE2 Trans hUC-MSCs. b) Full length blots of representative sample Lung tissue. iBright™ Prestained Protein Ladder (LC5615) was used as the molecular weight marker.


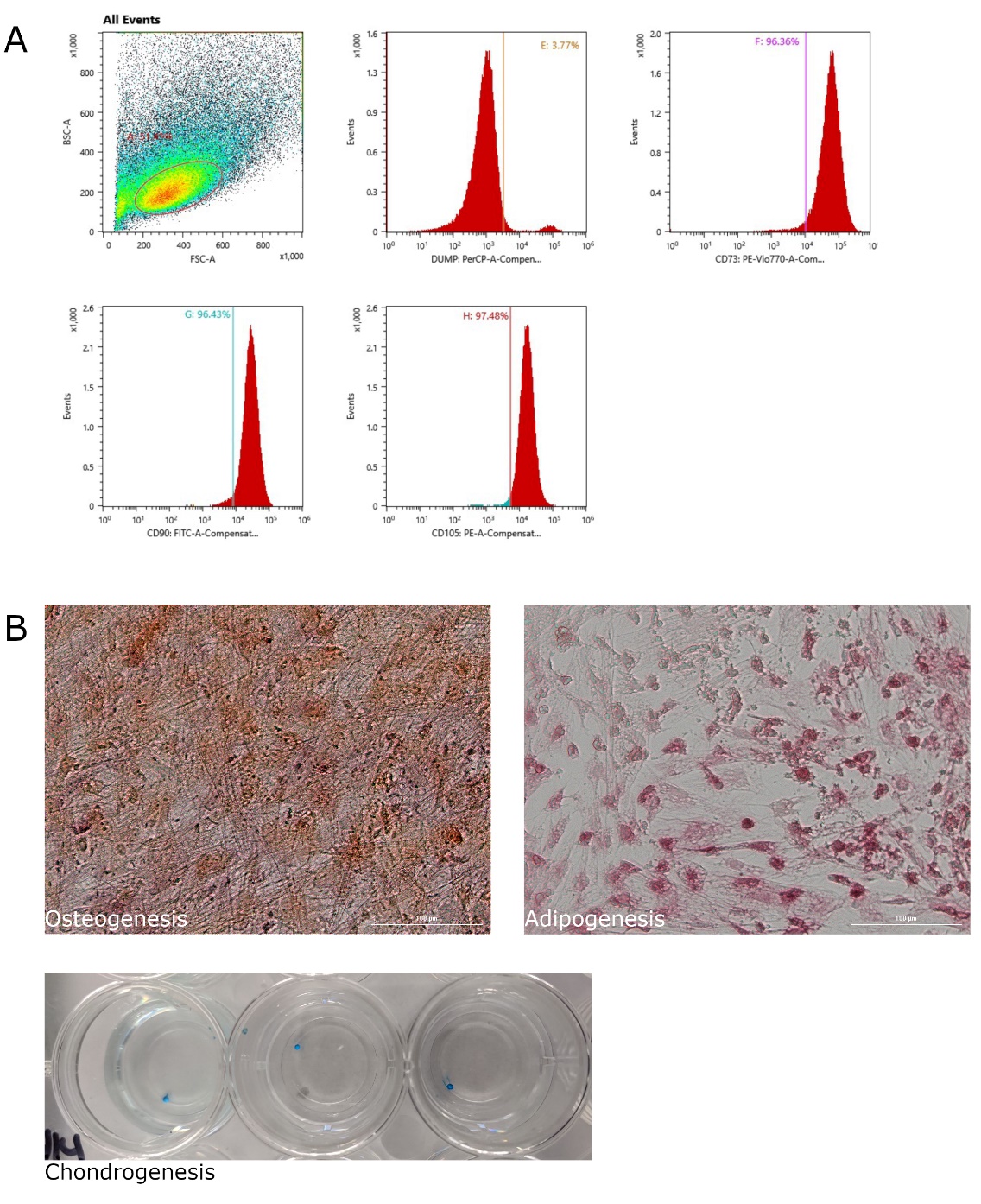


**a)**

**b)**

**Supplemental Figure 2. Immunophenotype and trilineage differentiation of hUC-MSCs lines**. a) Representative figures of flow cytometry analysis showing lower percentages of cells positive for CD14, CD20, CD34 and CD45 (DUMP:PreCP) and higher percentages of CD73 (PE-Vio770), CD90 (FITC) and CD105 (PE). b) Representative staining. Multipotency/Trilineage differentiation of hUC-MSCs was assayed after culture in specific medium for the osteogenic, adipocyte and chondrogenic differentiation. Specific extracellular matrix components were stained using Alzarin Red for osteoblasts, adipocytes were detected with Oil Red coloration of lipid droplets, and chondrocytes with alcian Blue.
